# A Proposal for a Noxious Stimuli-Free, Moderate-Intensity Treadmill Running Protocol to Improve Aerobic Performance in Experimental Research on Rats

**DOI:** 10.3390/metabo14100534

**Published:** 2024-10-04

**Authors:** Gilmara Gomes de Assis, Elda Olivia Nobre de Souza, Paulo Francisco de Almeida-Neto, Halil İbrahim Ceylan, Nicola Luigi Bragazzi

**Affiliations:** 1 Department of Odontology, School of Dentistry, Sao Paulo State University, Araraquara 14801-385, SP, Brazil; elda.nobre@unesp.br; 2 Health Sciences Center, Federal University of Rio Grande do Norte, CCS-UFRN, Natal 59078-970, RN, Brazil; 3 Physical Education and Sports Teaching Department, Faculty of Sports Sciences, Atatürk University, 25240 Erzurum, Turkey; halil.ceylan@atauni.edu.tr; 4 Laboratory for Industrial and Applied Mathematics (LIAM), Department of Mathematics and Statistics, York University, Toronto, ON M3J 1P3, Canada; robertobragazzi@gmail.com; 5 Human Nutrition Unit (HNU), Department of Food and Drugs, Medical School, University of Parma, 43125 Parma, Italy

**Keywords:** treadmill running, aerobic exercise, animal research, animal models, rat, fitness

## Abstract

**Background/Objectives:** Animal models can help understand human physiological responses, including the response to exercise and physical activity. However, many of these models incorporate noxious stimuli for various scientific purposes. We propose a noxious stimuli-free treadmill running training program for *Rattus norvegicus* species to study adaptations to aerobic exercise. **Methods:** In this study, rats were randomly allocated to training (*n* = 20) and sedentary (*n* = 20) groups. The training group underwent a program consisting of 30–50 min of treadmill running at 60% intensity, three times per week for 8 weeks. Maximum speed tasks (Tmax) were conducted to determine, adjust, and evaluate changes in fitness conditions. The rats had one week of familiarization with the treadmill, and a rubber ball was used at the back wall of the lane as a painless stimulus to encourage running. All assessments were conducted by two independent researchers in a double-blind manner, with data analysis conducted by a third-blind investigator. **Results:** A significant effect of time (η^2^_p_ = 0.430, *p* < 0.001, large effect) could be found, showing differences between Tmax1 and Tmax2, and between Tmax1 and Tmax3 in both groups. The training group significantly outperformed the sedentary group (η^2^_p_ = 0.266, *p* < 0.001, large effect). There was a significant interaction between time and condition (η^2^_p_ = 0.152, *p* < 0.001, large effect). **Conclusions:** The proposed moderate-intensity treadmill running program could effectively differentiate between trained and sedentary conditions within both the short period of 4 weeks and the extended period of 8 weeks. This protocol can be used as a model for running on a treadmill for *Rattus norvegicus* species without the use of noxious stimuli.

## 1. Introduction

Animal models—from murine to primate, porcine, and aquatic models (e.g., zebrafish)—represent a fundamental approach in pre-clinical studies, being crucial for understanding physiological responses, disease physiopathology, and developing novel treatments [1,2]. However, when it comes to behavioral research, the predictive power of animal experiments for human outcomes is debatable [3,4]. This issue often arises due to methodological flaws and the lack of standardized protocols, leading to subjectivity in interpreting results [3,4]. These factors can be particularly critical in developing countries, where limited resources and lower education levels may impact both scientific and ethical requirements, which are recognized as key factors influencing the proper use of animals in research [5].

Among the various animal models, rodents are particularly valuable for pre-clinical investigations due to their genomic similarity to *Homo sapiens*, as well as their manageable size and low maintenance costs [6]. The existing body of scholarly evidence built through research using animal models and exercise interventions in rodents has exponentially increased over the last few decades. Major achievements in clinical research would not have been possible without these animal studies. However, studies incorporating exercise protocols, such as “running on a treadmill” [5,7], are typically found in specific scientific fields such as neuroscience and pathology and are relatively scarce in exercise science.

From a physiological perspective, treadmill running for rodents offers significant advantages for investigating basic molecular mechanisms, biochemical and biophysical systems, and responses across species. Running is a natural activity for these animals, and the speed (intensity) and volume (duration) on the treadmill are controllable variables, allowing for precise workload calculations. Treadmill running protocols are, therefore, pivotal tools to validate findings from in vitro experiments and explore therapeutic strategies that may pose potential risks in pre-clinical research [5,7].

The proper use of rodents in exercise research protocols is a current topic of discussion. Ethics councils emphasize the importance of animal welfare, advocating for the prevention of suffering. Simultaneously, standardizing exercise science protocols and study designs is crucial to ensure reproducibility and minimize unnecessary experiment duplication and resource waste. However, there is often a lack of transparency in reporting research methods involving running exercise protocols in the scientific literature. Garrigos and colleagues [8] highlighted that many scientific papers do not provide sufficient data to guarantee experimental reproducibility and enable comparisons of research outputs.

Running protocols using rodent models are employed to compare physiological and pathophysiological parameters between experimental conditions and controls and to investigate changes in biological systems and molecular mechanisms in response to different metabolic efforts. Rodents serve as key models that allow for the assessment of critical tissues and organs, such as the brain, using techniques the invasiveness of which would be incompatible with human research [9,10,11]. In the last years, the use of treadmill running programs has gained popularity due to their advantages for exploring physiological adaptations and responses in rat models, including metabolism [5,7].

Traditionally, researchers have used electric shock as a “motivational stimulus” for rats in treadmill running protocols [12]. In a recent study by Sadri and colleagues [13], rats gradually achieved continuous running at a moderate speed with the use of mild electric shock as a motivational stimulus, following a brief familiarization period with the treadmill. In contrast, Burghardt and colleagues [14] demonstrated that rats increased their running speed and duration on the treadmill without the use of electric shocks [14]. However, while both studies identified differences in biochemical and behavioral parameters between trained and sedentary animals, they failed to provide detailed data on the animals’ fitness conditions (i.e., sedentary versus trained). The absence of this critical information on fitness parameters and protocol specifics hampers the ability to reproduce the experiments and conduct a meaningful comparative analysis of the rats’ performance, both with and without exposure to noxious stimuli.

Further, the physiological regulation of metabolism involves the systemic action of hormones involved in several stress-related response mechanisms, and stress factors can activate the “fight-or-flight” system and the release of stress hormones [15,16]. This implies that the physiological regulation of the rat’s performance during treadmill running exercise can be affected to the point that metabolic stressors might be altered in the animal’s organism [17,18,19].

When proposing guidelines for treadmill running protocols, it is essential to observe a series of principles. From an ethical standpoint, scholars should adhere to the so-called three R’s principles (Replacement, Reduction, and Refinement) [20,21], first laid out in 1959 by the Universities Federation for Animal Welfare (UFAW) scholars Russell and Burch. It is important to consider several criteria as well, including the natural characteristics of the animal, such as social behavior, anatomy, and genetic specificity, among others. When reporting the findings, according to the “Animal Research: Reporting of In Vivo Experiments” (ARRIVE) Guidelines 2.0 [22,23], details should be provided about (i) the study design, (ii) the sample size employed, (iii) inclusion and exclusion criteria, (iv) methods of randomization, (v) blinding/masking, (vi) outcome measures, (vii) statistical methods, (viii) experimental animals, (ix) experimental procedures, and (x) results. These ten core items ensure that the minimum necessary information is provided for assessing the reliability of the study, ultimately advancing scientific knowledge and improving animal welfare [22,23,24].

Taking all these factors into account can minimize the risk of biological/physiological and methodological biases, ensuring that animal research is not wasted, and the findings are meaningful, with animals experiencing “real” exercise rather than stress [5,7,22,23,24].

Traditional methods for investigating physical activity in animal research often involve harmful stimuli, such as electric shocks, to ensure compliance with exercise regimens. In this context, using stressful stimuli, such as low-intensity electric shocks via the touch of a metal grid, to keep animals running on a treadmill can introduce artifacts and confounding factors in scientific investigations [7]. Additionally, such stimuli can have negative effects on animals’ health and well-being and may obscure the true impact of exercise on health and aerobic capacity [25,26]. Recent advancements in research methodologies emphasize the use of non-noxious stimuli to explore physical activity in animal models. For example, using gentle prodding or placing a rubber ball behind the rats as a motivational stimulus has proven effective without causing undue stress. These noxious stimuli-free protocols enhance animal welfare and ensure the reliability and reproducibility of experimental data by minimizing stress-induced variability [27,28].

Furthermore, the relationship between aerobic capacity and the use of harmful stimuli in research is crucial for ensuring valid and ethical experimental outcomes. High aerobic capacity is associated with numerous health benefits, which can be significantly influenced by the presence of stressors. Noxious stimuli can alter stress hormone levels, immune responses, and overall behavior, potentially skewing the results of studies aimed at assessing aerobic capacity and its associated benefits [27]. Furthermore, high aerobic capacity can influence the animal’s ability to cope with and recover from various harmful stimuli, such as peripheral nerve injuries. For instance, recent studies demonstrated that rats with low aerobic capacity exhibit delayed recovery and disrupted sleep patterns following chronic peripheral neuropathy, suggesting that low aerobic fitness may exacerbate the negative consequences of such insults. These findings further illustrate the relationship between higher aerobic capacity and the animal’s ability to cope with various harmful stimuli [29,30]. Similarly, another recent investigation into the transcriptional adaptability of rats revealed that those with higher aerobic capacity showed enhanced responses to acute stressors, indicating that aerobic fitness may confer protective benefits against harmful stimuli [30].

Considering the studies mentioned above, using non-noxious methods to investigate the impact of exercise on rats running on treadmills during research interventions allows for a clearer assessment of the intrinsic benefits of high aerobic capacity without the confounding effects of stress. This approach aligns with good ethical research practices and ensures that the observed improvements in health and performance are genuinely attributable to aerobic training rather than stress-induced artifacts. Additionally, understanding the relationship between aerobic capacity and the animal’s response to harmful stimuli is crucial for developing targeted interventions and improving overall health outcomes [29].

The objective of the present study was to investigate the feasibility of implementing a treadmill-running training protocol for *Rattus norvegicus* without using electric shocks or painful stimuli. More specifically, we aimed to develop a low-to-moderate intensity treadmill running protocol that is reliable and reproducible and that can serve as a framework for noxious stimuli-free running training programs at different intensities.

## 2. Materials and Methods

**Figure 1:**
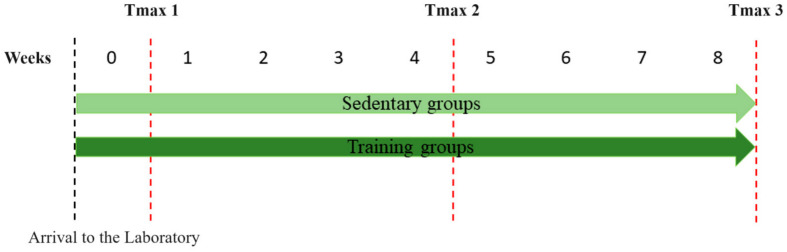
Study design. Tmax: Maximum speed performance on a treadmill. Tmax1; after one week of familiarization (week 0—adaptation), Tmax2; after 4 weeks of training, and Tmax3; after 8 weeks of training.

A total of 40 mices (*Rattus norvegicus*, Holtzman strain) were involved in the development of the treadmill running training program, with an equal split between training and sedentary groups. The training was conducted at a low-to-moderate intensity of 60% of maximum capacity (Figure 1).

All animals were obtained from the central Bioterium of São Paulo State University (UNESP), Araraquara, Brazil, under the permission granted by the local Ethical Committee for Animals (CEUA 20/2022) of the School of Dentistry, UNESP, Araraquara, Brazil. The animals were housed in Bioterium 2 of the School of Dentistry, maintained in a controlled environment with a temperature of 22–23 °C and a 12:12 h light/dark cycle. The running sessions were performed on a rodent treadmill manufactured by AVS Projects, between 8 a.m. and 11 a.m.

Rats, approximately 10 weeks old and weighing around 188 g at the time of arrival at the laboratory (as depicted in Figure 1), underwent a 1-week familiarization period (week 0) with the treadmill. This familiarization involved three slightly different adaptation protocols (A, B, and C), varying in the number of sessions and speed range. The goal was to determine whether the familiarization regime would affect the spontaneous running behavior of the animals during the maximum speed evaluation.

Group A, consisting of 42% of rats, followed Protocol A: namely, 8 sessions of 5 to 8 min of walking on a treadmill at a speed of 3 to 6 m/min over 8 days. Group B, consisting of 31% of rats, followed Protocol B: 5 sessions of 5 to 6 min of walking on a treadmill at a speed of 4 to 8 m/min over 8 days. Finally, Group C, consisting of 27% of rats, followed Protocol C: 5 sessions of 5 to 6 min of walking on a treadmill at a speed of 3 to 6 m/min over 8 days. After the familiarization period, all animals underwent a maximum speed test on a treadmill (Tmax). Subsequently, the exercise intensity was adjusted after a second Tmax assessment (Tmax2) before the 5th week of the training program to ensure it remained within the moderate intensity zone.

Statistical analysis using the general linear model (GLM) (see Section 2.2 for further details) showed that the adaptation sessions had no significant effect on the Tmax results (η^2^_p_ = 0.005, *p* = 0.441).

### 2.1. Tmax—The Treadmill Running Speed Performance Test

The animals were individually placed on the treadmill for 30 s before the start, with a cardboard cover obscuring about one-third of the treadmill to create a darker section where the rats should run. Importantly, no electrical shock or sound stimuli were necessary for this protocol. To help the animals keep pace with the treadmill speed and minimize stop-and-go behavior, a small rubber ball was placed at the tail end of the treadmill line to prevent the animals from reaching the end of the treadmill line with their lower back when they were distracted or exhausted. The maximum speed test (Tmax) began at a speed of 5 m/min for 3 min, with increments of 5 m/min applied every 3 min until the animals could no longer keep pace with the treadmill. It was assumed that the animal had achieved its maximum speed performance when it could no longer run adequately and touched the end of the treadmill line with its back for more than 3 s, using the back wall for support. At the first sign of this behavior, the evaluator repositioned the animal by gently pushing its hind legs. When the animal was unable to maintain the running pattern and remained at the back wall of the treadmill, the evaluator terminated the test [31].

The duration and speed of the tests were recorded to determine the stage (in meters per minute) and time (in seconds) at which the animals reached Tmax. Additionally, the absolute workload was calculated for each animal.

We employed the following mathematical model (Equation (1)) [32]:

(1) $$workload\;\left(J\right)=m\cdot g\cdot s\;\cdot \sin\;(\theta )\cdot t$$

where *m* is the body mass in kg; *g* is the gravitational acceleration (9.8 m/s^2^); *s* is the speed in m/min; *θ* is the treadmill inclination angle in degrees; and *t* is the time spent on each step. Then, we determined the relative workload by dividing the absolute workload by the animal’s body weight. To verify the subjectivity of the Tmax, two independent evaluators assessed the Tmax across the groups, as follows:

Evaluator 1: Group A—Tmax1, Tmax2, Tmax3; Group B—Tmax1
Evaluator 2: Group B—Tmax2, Tmax3; Group C—Tmax1, Tmax2, Tmax3

Statistical analyses using a GLM (see Section 2.2 for further details) confirmed that the Tmax assessment conducted by the different evaluators (1 or 2) did not have a significant effect on the interventions (η^2^_p_ = 0.003, *p* = 0.2).

After Tmax1, the animals were randomly assigned to either an 8-week running training program or a sedentary control group, as described in Table 1. For randomization, the animals were tagged with numbers, and a raffle determined their assignment to either the ‘training’ or ‘sedentary’ groups. The training program intensity was set at 60% of the animals’ maximum speed, calculated from the average time and speed recorded during the Tmax test of the training group animals. The detailed 8 week low-intensity treadmill running program is outlined in Table 1. During the training period, sedentary group animals underwent a familiarization routine twice a week. All tests and training sessions were conducted in a completely silent room. A second Tmax test (Tmax2) was administered to all animals at the end of the 4th week, and a third Tmax test (Tmax3) at the end of the 8th week.

### 2.2. Statistical Analyses

Data analyses were performed in a double-blinded manner by an external research collaborator. Descriptive statistical analysis followed the recommendations by Mishra et al. [33]. The normality of variable distributions was verified using the Shapiro-Wilk test, examining asymmetry and kurtosis values between −1.96 and 1.96, and quantile-quantile (Q-Q) plots. Student’s *t*-test was used for sample characterization. Before comparisons, including the effect of time (Tmax1, Tmax2, and Tmax3), Levene’s test verified the homogeneity of variance across samples (Tmax), and Mauchly’s test assessed the statistical assumption of sphericity. The GLM with Bonferroni post-hoc tests was used for comparative analyses of time (Tmax1 × Tmax2 × Tmax3) and conditions (exercise and sedentary groups). The effect size of differences was calculated using partial eta squared (η^2^_p_), with magnitudes classified as small (<0.06), medium (0.06 to 0.14), and large (>0.14) [34]. GLM was also used to verify the effect of familiarization on groups (A, B, and C), the evaluator for the group (evaluator 1 and evaluator 2), and the number of adaptation sessions (five and eight sessions). Finally, linear regression analyses (considering the conditions [training and sedentary groups]) were conducted to assess the potential contribution of performance in test 1 to the results of tests 2 and 3 (for the variables Tmax, absolute workload, and relative workload).

A significance level of *p* < 0.05 was applied to all statistical analyses, which were performed using Jamovi^®^ software (Version: 2.3.18, Sydney, Australia).

## 3. Results

**Figure 2:**
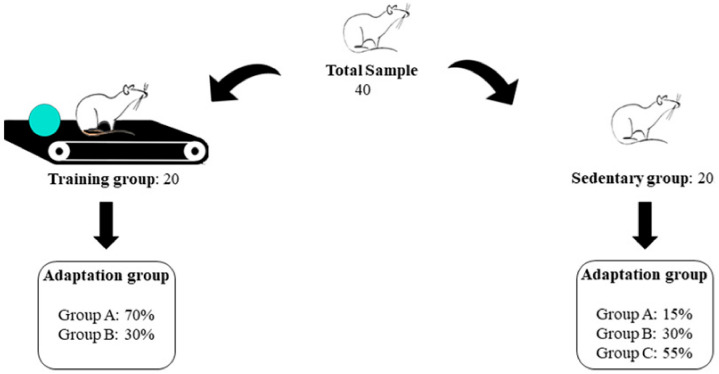
Randomization and group formation. Group A—protocol: 8 sessions of 5 to 8 min walking on the treadmill at the speed of 3 to 6 m/min for 8 days. Group B—protocol: 5 sessions of 5 to 6 min walking on the treadmill at the speed of 4 to 8 m/min for 8 days. Group C—protocol: 5 sessions of 5 to 6 min walking on the treadmill at the speed of 3 to 6 m/min for 8 days.

Figure 2 depicts the randomization process for forming the training and sedentary groups, as well as the familiarization protocols (A, B, and C).

No significant differences in the animals’ body weight between the familiarization groups (*p* > 0.05) could be computed at Tmax1, while differences could be found at Tmax2 and Tmax3 (see Table 2). No significant effect of familiarization (adaptation protocols A, B, or C) on the results analyzed in this study could be found (η^2^_p_ = 0.041, *p* = 0.185, small effect) (Figure 2).

Table 3 shows the comparisons between the groups considering the effect of time (Tmax1 versus Tmax2 versus Tmax3), condition (training versus sedentary groups), and interaction between time and condition.

A significant effect of time (F = 43.3, η^2^_p_ = 0.430, *p* < 0.001, large effect) was found, indicating a difference between Tmax1 and Tmax2, and between Tmax1 and Tmax3, in both groups for all the variables (Table 3). No significant difference could be found between Tmax2 and Tmax3 for all the variables. There was a significant difference in terms of the experimental condition (F = 41.3, η^2^_p_ = 0.266, *p* < 0.001, large effect), favoring the training group over the sedentary group in Tmax2 and Tmax3 for all the variables, and a significant interaction between time and condition (F = 10.2, η^2^_p_ = 0.152, *p* < 0.001, large effect) for all the variables under study.

Finally, linear regression analyses (considering the experimental condition [training and sedentary groups]) indicated that the results of Tmax1 did not have a significant effect on the results of Tmax2 and Tmax3. This indicates that changes at Tmax2 and Tmax3 were likely due to the training program (treadmill time: R^2^ = 0.42; workload: R^2^ = 0.38; and relative workload: R^2^ = 0.35).

## 4. Discussion

The proposed noxious stimuli-free, low-to-moderate-intensity, 8-week treadmill running protocol for *Rattus norvegicus* effectively increased fitness in young to adult animals within just 4 weeks and maintained elevated fitness levels over the full period of 8 weeks. The Resource Book for the Design of Animal Exercise Protocols [31] notes that treadmill running may not represent a rat’s natural physical activity pattern if construed as forced exercise or if noxious stimuli (e.g., electric shocks or bursts of high-pressure air) are used. Additionally, rats display a natural “stop-and-go” running behavior, which on a treadmill can be misinterpreted as tiredness, exhaustion, or fear, thereby increasing stress when noxious stimuli are present [31]. In our protocol, familiarization was crucial to avoid the “stop-and-go” behavior and to enable the performance of a maximum effort test and the progression of training sessions without using noxious stimuli.

Despite the abundance of scientific experiments involving treadmill running in rodent models, practical guides for protocols that can be replicated in further research are scarce or nonexistent in the literature. Our results demonstrate that the proposed 8-week treadmill running protocol is effective for improving fitness in *Rattus norvegicus*. Different investigators successfully replicated and conducted the protocol, indicating its feasibility. It could significantly enhance the fitness condition of young adult rats within 4 weeks and maintain this improvement for an additional 4 weeks. Previous studies on treadmill running in rats have also identified this plateau behavior.

In the study by Wisløff et al. [35], who measured maximal oxygen uptake (VO_2max_) in Sprague-Dawley rats using a controlled treadmill running protocol, a plateau in VO_2max_ gains was observed after seven weeks of training. The rats ran for 2 h per day, 5 days a week, alternating between 8 min intervals at 85–90% of their VO_2max_ and 2 min intervals at 50–60% VO_2max_. The training regimen resulted in a 60–70% increase in VO_2max_ compared to sedentary controls.

Similarly, Teixeira-Coelho et al. [36] found a plateau in speed gains from the fourth week onwards in an 8-week treadmill running protocol, despite increments of up to 225% of maximum speed during the training program. This highlights the significant role of intensity in the progression and timing of fitness gains in rats, akin to humans, despite physiological differences between species. The authors concurred that improved running economy likely contributes to the plateau observed in fitness variables when rats undergo long-term steady training programs.

Investigations into exercise physiology in rodents date back nearly half a century. In a foundational study [37] on exhaustion mechanisms in rats, researchers explored whether mitochondrial disruption and reduced oxidative metabolism were key contributors to muscle fatigue. Biomarkers of oxidative capacity and mitochondrial integrity were examined in the skeletal muscles of trained rats. The animals underwent moderate-to-high intensity training, after which the rats were subjected to an exhaustive running protocol, continuing until they could no longer right themselves when placed on their backs. The study revealed that muscle glycogen was significantly depleted, but mitochondrial integrity remained intact. These findings suggest that mitochondrial disruption is not a primary cause of fatigue or exhaustion, pointing instead to other factors as the culprits.

According to such studies, it is of crucial importance to investigate the properties of skeletal muscles, focusing on the proportions and characteristics of slow-twitch oxidative (Type I), fast-twitch oxidative glycolytic (Type IIa), and fast-twitch glycolytic (Type IIx) muscle fibers. These different muscle fiber types contribute to changes in oxidative capacity in response to exercise. Some studies [37,38] showed that the medial and lateral gastrocnemius muscles are similar regarding the populations of Type I and IIa fibers, while the soleus muscle contains a higher proportion of Type I fibers compared to the gastrocnemius. After high-intensity training, analyses [37,38] revealed that, while Type I fibers showed an increase in oxidative capacity across all training programs, the increase in oxidative capacity for Type IIx fibers depended on the intensity and was never as substantial as that observed in Type IIa fibers.

Dudley et al. [39] investigated the influence of exercise intensity and volume on muscle fibers’ oxidative capacity response using various training intensities in rats. Analyses of Type IIa and Type IIx fibers from the superficial and deep *vastus lateralis* and Type I fibers from the soleus showed that changes in cytochrome c levels were proportional to the exercise volume. Maximal adaptive changes were observed at each running intensity, with higher intensities requiring shorter volumes to achieve peak muscle oxidative capacity. The study also identified a threshold for Type IIx fibers below which no increases in cytochrome c concentrations occurred. In these fibers, cytochrome c levels began to increase and rose exponentially. In contrast, Type IIa fibers exhibited a plateau in cytochrome c increase. These findings imply that different muscle fiber types contribute differentially to the muscle’s adaptive response to endurance exercise. Like humans, rats physiologically adapt to the metabolic demands imposed by exercise and achieve fitness gains more efficiently as exercise intensity increases, provided the intensity is not excessive [40,41].

Additionally, in our study, habituation to the treadmill was crucial to avoid biases in the performance test and to apply the protocols without using noxious stimuli. These protocols were repeated to establish a standardized routine, which is scarce in the literature and often leads to wasted time and animal lives.

In our protocol model, the different adaptive routines tested before Tmax1 showed similar effects on Tmax1 performance. A small number of adaptation sessions—such as 5 sessions of walking at speeds of 3 to 6 m/min for 5 to 6 min—is sufficient to prepare the animals for Tmax evaluation.

Despite the relevant findings, the present study has limitations, such as not analyzing physiological parameters such as cortisol levels or the behavior of the animals during the intervention period. Such analyses would help determine if the proposed protocol significantly affected physiological and behavioral stress levels through the painless approach used.

## 5. Conclusions

The 4-week low-intensity training (60% maximum speed) was sufficient for the rats to achieve a fitness level significantly higher than that of non-running laboratory rats. This training regime maintained this improvement for at least another 4 weeks. A rubber ball placed at the tail end of the treadmill served as a corrective stimulus, keeping the animals running and preventing stop-and-go behavior, thus eliminating the need for noxious stimuli. This training protocol can be replicated or used as a basis for further running training protocols.

Considering the importance of animal studies in pre-clinical research and the 3 R’s principle, establishing standardized protocols helps minimize the number of animals used in experiments and reduces research costs.

## Figures and Tables

**Table 1:** Treadmill running protocol for 8 weeks of exercise at 60% of Tmax intensity.

(a) First four weeks of treadmill running for *Rattus norvegicus*								
	**Week 1**		**Week 2**		**Week 3**		**Week 4**	
	% Speed	Time (min)	% Speed	Time (min)	% Speed	Time (min)	% Speed	Time (min)
Day 1	40%	2	40%	4	40%	5	40%	5
	50%	3	50%	12	50%	15	50%	13
	60%	5	60%	19	60%	20	60%	24
	30%	2	30%	5	30%	5	30%	3
Day 2	40%	2	40%	5	40%	5	40%	5
	50%	8	50%	17	50%	20	50%	20
	60%	7	60%	21	60%	32	60%	30
	30%	3	30%	5	30%	3	30%	5
Day 3	40%	3	40%	2	40%	4	40%	5
	50%	6	50%	13	50%	13	50%	20
	60%	13	60%	20	60%	24	60%	30
	30%	3	30%	5	30%	4	30%	5
Day 4	40%	3	40%	3	40%	3	40%	4
	50%	9	50%	21	50%	21	50%	10
	60%	15	60%	26	60%	33	60%	20
	30%	3	30%	5	30%	3	30%	2
Day 5	40%	3	40%	4	40%	3	Tmax2	
	50%	11	50%	23	50%	19		
	60%	18	60%	28	60%	35		
	30%	4	30%	5	30%	3		
(b) Second four weeks of treadmill running for *Rattus norvegicus*								
	**Week 5**		**Week 6**		**Week 7**		**Week 8**	
	% Speed	Time (min)	% Speed	Time (min)	% Speed	Time (min)	% Speed	Time (min)
Day 1	24%	2	24%	2	32%	2	32%	3
	32%	2	32%	2	40%	3	44%	3
	40%	5	44%	8	44%	3	52%	6
	44%	8	52%	12	52%	12	60%	32
	52%	9	60%	15	60%	20	32%	4
	60%	2	32%	6	64%	2	-	-
	44%	12	-	-	32%	3	-	-
	24%	5	-	-	-	-	-	-
Day 2	32%	5	32%	3	24%	3	32%	3
	40%	7	40%	3	36%	3	44%	3
	44%	19	44%	6	44%	3	52%	12
	52%	23	52%	18	52%	14	60%	36
	32%	6	60%	22	60%	30	32%	4
	-	-	32%	5	32%	6	-	-
	-	-	-	-	-	-	-	-
Day 3	24%	2	32%	3	32%	3	32%	3
	32%	3	40%	3	44%	3	44%	3
	44%	5	44%	3	52%	6	52%	6
	52%	20	52%	10	60%	26	60%	10
	60%	5	60%	21	64%	4	64%	2
	40%	5	32%	5	32%	3	68%	2
	32%	5	-	-	-	-	60%	10
	-	-	-	-	-	-	32%	4
Day 4	32%	5	32%	3	24%	3	32%	3
	40%	5	40%	3	36%	3	40%	5
	44%	8	44%	3	44%	3	44%	8
	52%	12	52%	10	52%	8	52%	17
	60%	10	60%	28	60%	38	60%	22
	32%	5	32%	6	32%	5	32%	5
	-	-	-	-	-	-	-	-
Day 5	32%	2	32%	3	32%	3	Tmax3	
	40%	3	40%	3	40%	3		
	44%	5	44%	3	44%	3		
	52%	20	52%	10	52%	9		
	60%	15	60%	30	60%	30		
	32%	5	32%	6	64%	6		
	-	-	-	-	32%	3		

**Table 2:** Sample characterization regarding the number of adaptation sessions and body weight at the time of Tmax.

Variables	Training (*n* = 20)	Sedentary (*n* = 20)	*p*-Value
Adaptation sessions (n)	6.5 ± 2.3	5.8 ± 1.8	0.291
Weight (g)—Tmax1	261.0 ± 13.1	256.0 ± 13.6	0.244
Weight (g)—Tmax2	378.0 ± 14.0	357.0 ± 19.8	0.0004
Weight (g)—Tmax3	422.0 ± 15.9	402.0 ± 24.5	0.004

Data processing: Descriptive statistics and Student’s independent *t*-test. Tmax: Maximum speed performance on a treadmill.

**Table 3:** A general linear model comparing the training and sedentary groups at the time of Tmax.

Variable	Time:	Tmax1		Tmax2		Tmax3		General Linear Model (Effects)		
	Condition:	Training	Sedentary	Training	Sedentary	Training	Sedentary	Time	Condition	Interaction
		Mean ± SD						*p*-Value (η^2^_p_)		
Treadmill Time (s)		529.0 ± 88.8	523.0 ± 78.1	831.0 ± 1146 †⸸	625.0 ± 133.3 †	754.0 ± 91.1 †⸸	608.0 ± 94.2 †	<0.001 (0.430)	<0.001 (0.266)	<0.001 (0.152)
Workload (J)		202.0 ± 65.9	191.8 ± 55.7	687.5 ± 184.0 †⸸	375.6 ± 151.7	651.9 ± 166.7 †⸸	399.3 ± 121.7	<0.001 (0.591)	<0.001 (0.351)	<0.001 (0.201)
Relative Workload (J/kg)		0.8 ± 0.3	0.7 ± 0.2	1.8 ± 0.5 †⸸	1.0 ± 0.4 †	1.5 ± 0.4 †⸸	1.0 ± 0.3 †	<0.001 (0.389)	<0.001 (0.282)	<0.001 (0.159)

Tmax: Maximum speed performance on a treadmill. †: Significant difference in relation to Tmax1 (*p* < 0.05). ⸸: Significant difference between the sedentary group and the training group (*p* < 0.05).

## Data Availability

The original contributions presented in the study are included in the article, further inquiries can be directed to the corresponding authors.
